# Newborn sex-specific transcriptome signatures and gestational exposure to fine particles: findings from the ENVIR*ON*AGE birth cohort

**DOI:** 10.1186/s12940-017-0264-y

**Published:** 2017-06-05

**Authors:** Ellen Winckelmans, Karen Vrijens, Maria Tsamou, Bram G. Janssen, Nelly D. Saenen, Harry A. Roels, Jos Kleinjans, Wouter Lefebvre, Charlotte Vanpoucke, Theo M. de Kok, Tim S. Nawrot

**Affiliations:** 1 Centre for Environmental Sciences, Hasselt University, Agoralaan gebouw D, B-3590 Diepenbeek, Belgium; 2 Louvain Centre for Toxicology and Applied Pharmacology (LTAP), Université catholique de Louvain, Brussels, Belgium; 3 Department of Toxicogenomics, Maastricht University, Maastricht, The Netherlands; 4 Environmental Risk and Health, Flemish Institute for Technical Research (VITO), Mol, Belgium; 5 Belgian Interregional Environment Agency (IRCEL), Brussels, Belgium; 6 Department of Public Health & Primary Care, Leuven University, Kapucijnenvoer 35, 3000 Leuven, Belgium

**Keywords:** Ambient air pollution, Particulate matter, Microarray analysis, Fetal, Sex

## Abstract

**Background:**

Air pollution exposure during pregnancy has been associated with adverse birth outcomes and health problems later in life. We investigated sex-specific transcriptomic responses to gestational long- and short-term exposure to particulate matter with a diameter < 2.5 μm (PM_2.5_) in order to elucidate potential underlying mechanisms of action.

**Methods:**

Whole genome gene expression was investigated in cord blood of 142 mother-newborn pairs that were enrolled in the ENVIR*ON*AGE birth cohort. Daily PM_2.5_ exposure levels were calculated for each mother’s home address using a spatial-temporal interpolation model in combination with a dispersion model to estimate both long- (annual average before delivery) and short- (last month of pregnancy) term exposure. We explored the association between gene expression levels and PM_2.5_ exposure, and identified modulated pathways by overrepresentation analysis and gene set enrichment analysis.

**Results:**

Some processes were altered in both sexes for long- (e.g. DNA damage) or short-term exposure (e.g. olfactory signaling). For long-term exposure in boys neurodevelopment and RhoA pathways were modulated, while in girls defensin expression was down-regulated. For short-term exposure we identified pathways related to synaptic transmission and mitochondrial function (boys) and immune response (girls).

**Conclusions:**

This is the first whole genome gene expression study in cord blood to identify sex-specific pathways altered by PM_2.5_. The identified transcriptome pathways could provide new molecular insights as to the interaction pattern of early life PM_2.5_ exposure with the biological development of the fetus.

**Electronic supplementary material:**

The online version of this article (doi:10.1186/s12940-017-0264-y) contains supplementary material, which is available to authorized users.

## Background

Changes in the transcriptome biology during fetal development can contribute to disease susceptibility. The fetal developmental period is known to be highly sensitive to environmental stressors causing alterations at different omic levels which may result in increased risk of disease in adulthood [1–3]. It has been hypothesized that specific transcriptome profiles in response to gestational exposure to fine particulate matter (PM) may not only act as signatures of exposure but could also be potentially prognostic for exposure-related health outcomes later in life.

Several observational studies corroborated the relationship between PM air pollution and adverse birth outcomes, such as decreased fetal growth [4–6] and preterm birth [7, 8]. Furthermore, perinatal physiological parameters like newborn systolic blood pressure were found to be associated with PM exposure during gestation [9]. Gestational air pollution exposure may affect the fetus in two different ways: 1) indirectly, through mediation by inflammatory effects on the mother’s cardiorespiratory system and 2) directly, after translocation of (ultra)fine particles via the mother’s bloodstream to the placenta. Wick et al. demonstrated in an ex vivo human placental perfusion model that polystyrene particles with a diameter up to 240 nm are able to cross the placental barrier [10].

There is suggestive evidence that prenatal air pollution exposure may be linked to various adverse effects later in life such as cognitive and behavioral changes [11, 12], cancer [13, 14], and respiratory ailments [15, 16]. In addition, some studies reported sex differences in air pollution-related adverse health effects [17, 18]. Penaloza and colleagues [19] showed that sex-specific effects of prenatal exposure to environmental stressors are not only attributed to hormonal but also to chromosomal differences. Another study reported sex-specific associations between persistent organic pollutants and cord sex hormones [20].

PM air pollution is an omnipresent environmental risk factor for public health in large areas of the world, however, the impact of gestational exposure to PM air pollution on fetal transcriptome profiles has not been assessed so far. In order to elucidate potential molecular mechanisms underlying prenatal PM_2.5_-induced adverse health effects, we investigated sex-specific transcriptomic responses in cord blood as part of the early life exposome in the framework of the ENVIR*ON*AGE birth cohort.

## Methods

### Study population

Mother-child pairs were enrolled in the on-going ENVIR*ON*AGE birth cohort (ENVIRonmental influence *ON* early AGEing) following procedures previously approved by the Ethical Committee of Hasselt University and the East-Limburg Hospital (09/080 U;B37120107805) [21], and complies with the Helsinki declaration. All participating mothers provided written informed consent. Cord blood samples were collected along with perinatal parameters such as birth date, gestational age, newborn’s sex, birth weight and length. The mothers completed study questionnaires in the post-delivery ward to provide detailed information on maternal age, pre-gestational body mass index (BMI), maternal education, smoking status, alcohol consumption, place of residence, parity, and ethnicity of the newborn. Former-smokers were defined as those who had quit smoking before pregnancy. Smokers were those who continued smoking during pregnancy. Based on the native country of the newborn’s grandparents we classified his/her ethnicity as European-Caucasian when two or more grandparents were European, or as non-European when at least three grandparents were of non-European origin. We asked the mothers whether they consumed alcohol during pregnancy. Maternal education was coded as “low” (no diploma or primary school), “medium” (high school) or “high” (college or university degree).

The ENVIR*ON*AGE birth cohort had an overall participation rate of 61%. The current study is based on a representative subgroup of the ENVIR*ON*AGE birth cohort including 150 newborns recruited from South-East-Limburg Hospital in Genk (Belgium) born between Friday 1200 h and Monday 0700 h from March 20th 2010 until March 9th 2014. The general characteristics of the mother-child pairs did not differ from all births in Flanders as to maternal age, education, parity, sex, ethnicity, and birth weight (See Additional file 1: Table S1). Quality control of microarray data resulted in exclusion of four newborns. Of the remaining 146 newborns, we excluded four newborns for whom no prenatal exposure (lived outside the study area) were available. This resulted in a final sample of 142 mother-child pairs.

### Ambient PM_2.5_ exposure assessment

For each mother’s residential address, PM_2.5_ was calculated using a spatial temporal interpolation method (Kriging) taking into account land cover data obtained from satellite images (CORINE land cover data set) for interpolating the pollution data collected in the official fixed-site monitoring network in combination with a dispersion model (IFDM) using emissions from line sources and point sources [22–24]. This model chain provides daily PM_2.5_ values on a high resolution receptor grid. Overall, model performance was evaluated by leave-one-out cross-validation including 34 monitoring points for PM_2.5_. In our study area, the interpolation tool explained more than 80% of the temporal and spatial variability [24]. We defined two exposure windows of interest i.e. long-term (annual average before delivery) and short-term (last month of pregnancy) exposure. Annual averages before delivery were preferred to gestational exposure since annual averages are independent of season of blood sampling, an important predictor of gene expression [25]. Moreover, maternal PM_2.5_ exposure during the 3 months before conception may induce maternal changes that may indirectly affect conception and the fetus and is thus included in annual averages. One month was taken as a period reflecting short-term exposure. Complete information was obtained for the residential address during and before pregnancy. For those who moved during pregnancy (*n* = 19; 13.4%), we calculated the exposure allowing for the changes in address during this period.

Meteorological data including mean daily air temperature and relative humidity were measured at the federal official station and provided by the Belgian Royal Meteorological Institute (Brussels, Belgium). Apparent temperature was averaged over one week before delivery and categorized based on the 25th, 50th and 75th percentiles.

### RNA isolation

Total RNA was isolated from whole blood collected in Tempus tubes (ThermoFisher Scientific, Waltham, MA, USA) using the Tempus Spin RNA Isolation kit (Life Technologies, Paisley, UK) according to the manufacturer’s instructions. RNA yields were determined using the NanoDrop Spectrophotometer (Isogen Life Sciences, De Meern, the Netherlands) and the quality was checked on an Agilent 2100 Bioanalyzer (Agilent Technologies, Amstelveen, the Netherlands). Samples with RNA Integrity Number below 6 were excluded from further analysis. Samples were stored at −80 °C until further processing.

### Microarray preparation, hybridization and preprocessing

An aliquot of 0.2 μg total RNA was reverse-transcribed into cDNA, labeled with cyanine-3 following the Agilent one-color Quick-Amp labeling protocol (Agilent Technologies) and hybridized onto Agilent Whole Human Genome 8 × 60 K microarrays. Microarray signals were detected using the Agilent DNA G2505C Microarray Scanner (Agilent Technologies). Scan images were converted into TXT files using the Agilent Feature Extraction Software (Version 10.7.3.1, Agilent Technologies, Amstelveen, The Netherlands), which were imported in R 2.15.3 (http://www.r-project.org). An in-house developed quality control pipeline in R software was used to preprocess raw data as follows: local background correction, omission of controls, flagging of bad spots and spots with too low intensity, log_2_ transformation and quantile normalization using arrayQC. The R-scripts of the quality control pipeline and more detailed information on the flagging can be found at https://github.com/BiGCAT-UM/arrayQC_Module. Further preprocessing included removal of genes with more than 30% flagged data, merging of replicates based on the median, imputation of missing values by means of K-nearest neighbor imputation (K = 15) and correction for batch effects using an empirical Bayes method [26]. For genes represented by multiple probes, only the probe with the largest interquartile range was considered. The final dataset used for statistical analyses contained 16,844 genes.

### Data analysis

To study alterations in gene expression in association with long-term (one year before delivery) and short-term (one month before delivery) exposure, multivariable-adjusted linear regression was performed while accounting for gestational age, season of conception, averaged apparent temperature over the last week of pregnancy (categories: <4.4, 4.4–7.9, 7.9–14.1, >14.1 C°), parity (first, second, higher-order birth), maternal age, smoking status (never, past or current smoker), maternal education (lower secondary or less, higher secondary, higher education), ethnicity of the grandparents (European-Caucasian, yes or no), gestational age, pre-pregnancy BMI, newborn’s sex, long- or short-term PM_2.5_ exposure, and the interaction term between newborn’s sex and exposure. The interaction term was included in the models based on previous evidence suggesting differential responses between both sexes to environmental stressors during the perinatal period. Also at gene expression level, several animal studies [19, 27–30] and an epidemiological study of Hochstenbach and colleagues [2] observed sex-specific responses to prenatal environmental stress. For each sex, fold changes were calculated for an increase in long-term PM_2.5_exposure of 5 μg/m^3^ and for an increase of 10 μg/m^3^ in short-term PM_2.5_ exposure. A *p*-value smaller than 0.05 was considered significant. A principal component analysis was performed based on the significant genes (*p*-value <0.05) for long- and short-term exposure for both sexes. Partial correlation coefficients (R) were calculated between principal component scores and long- and short-term PM_2.5_ exposure.

In a sensitivity analysis, we additionally adjusted for white blood cell (WBC) counts and the percentage of neutrophils. However, due to blood clotting, data on these two variables were missing for 31 newborns. Normally, at birth the amount of WBCs ranges from 9 to 30 × 10^3^/μL. One newborn was excluded due to an outlying WBC count (>35 × 10^3^/μL). We assumed data is “at least missing at random”. Single stochastic regression imputation was performed in SAS using the FCS statement in proc MI. For the WBC counts and percentage of neutrophils, we included in the imputation model the covariates of the main model and, respectively the top three significant genes related to WBC counts and neutrophil percentage resulting from a complete case analysis.

### Pathway analysis by ConsensusPathDB

Genes significantly (*p* < 0.05) associated with PM_2.5_ exposure were uploaded into the Online Overrepresentation Analysis Tool ConsensusPathDB (http://consensuspathdb.org/) [31] of the Max Planck Institute for Molecular Genetics, to identify pathways associated with PM_2.5_ exposure. A *p*-value representing the pathway of smaller than 0.05 was considered significant.

### Gene set enrichment analysis

The GSEA (Gene Set Enrichment Analysis) software tool (MSigDB, version 5.0) [32, 33] was used to find pathways significantly correlated with PM_2.5_ exposure. Genes were ranked by the log_2_-fold change. Subsequently, an enrichment score was calculated reflecting the degree a pathway is enriched by highly ranked genes. The statistical significance was estimated using a gene set permutation test with false discovery rate (FDR) correction for multiple hypothesis testing.

Pathways with a q-value (FDR adjusted *p*-value) below 0.05 and *p*-value smaller than 0.005 were considered significant. Significant pathways were visualized using plug-in EnrichmentMap of cytoscape 3.2.0 software (http://cytoscape.org) [34]. An overlap coefficient of 0.5 was applied as similarity cutoff.

## Results

Table 1 shows demographic characteristics and perinatal traits of the mother-child group (*n* = 142). Mean maternal age was 29.3 (range: 18-42) years and mean (SD) pre-gestational BMI was 24.2 (4.6) kg/m^2^. Most women never smoked (*n* = 80), 36 women stopped smoking before pregnancy, whereas 26 mothers reported to continue smoking during pregnancy (on average 8.6 cigarettes/day). More than 80% of the mothers never used alcoholic beverages during pregnancy. The newborns, among them 76 girls (53.5%), had a mean gestational age of 39.7 weeks (range, 35.9–41.1) and comprised 70 primiparous and 59 secundiparous newborns. About 90% of the newborns were Europeans of Caucasian ethnicity and their mean (SD) birth weight was 3454 (431) g. Maternal exposure to PM_2.5_ over one year (long-term) and one month (short-term) preceding delivery averaged 16.0 (range: 11.8–20.6) and 13.3 (range: 6.5–34.8) μg/m^3^ respectively.

**Table 1:** Demographic characteristics of the study population and exposure (*n* = 142)

Characteristic	Mean (p10, p90) or *n* (%)
Mothers	
Age, yrs	29.3 (24.0, 34.0)
Pre-gestational BMI, kg/m^2^	24.2 (19.5, 30.5)
Education	
Low	15 (10.6%)
Medium	50 (35.2%)
High	77 (54.2%)
Smoking status	
Never-smoker	80 (56.3%)
Former-smokers	36 (25.4%)
Smokers	26 (18.3%)
Alcohol consumption	
No	119 (83.8%)
Occasionally	23 (16.2%)
Parity	
1	70 (49.3%)
2	59 (41.5%)
≥ 3	13 (9.2%)
Newborns	
Sex	
Boys	66 (46.5%)
Season at conception	
Winter	38 (26.8%)
Spring	40 (28.2%)
Summer	37 (26.1%)
Autumn	27 (19.0%)
Ethnicity	
European-Caucasian	124 (87.3%)
Gestational age, wks	39.7 (38.3, 41.1)
Birth weight, g	3454 (2910, 4045)
Exposure	
Long-term^a^ PM_2.5_, μg/m^3^	16.0 (13.9, 18.3)
Short-term^b^ PM_2.5_, μg/m^3^	13.3 (8.0, 21.4)
Weekly apparent temp, °C	8.9 (2.4, 16.5)

*p* percentile

^a^Annual average before delivery. ^b^Last month of pregnancy

A histogram of the percentage of genes associated with each of the covariates included in the model (*p*-value <0.05) is given in Additional file 1: Figure S1.

The effect of long-term gestational PM_2.5_ exposure (annual average before delivery) on gene expression in cord blood revealed major differences between girls and boys. A total of 1269 (7.5%) genes showed a significant interaction between fine particle air pollution and the sex of the newborn. For girls and boys, this study identified respectively 724 and 1358 genes which were significantly associated with long-term gestational PM_2.5_ exposure. Among these genes, 75 were differentially expressed in both boys and girls (see Additional file 1: Table S2). Additional file 1: Table S3 represents the top ten significant genes for boys and girls separately and their fold changes for a 5 μg/m^3^ increment in PM_2.5_ exposure.

Additional file 1: Figure S2A and B show the association of the first and second principal component score with long-term PM_2.5_ exposure for girls and boys respectively. Both principal components were significantly associated with long-term PM_2.5_ exposure in both girls (PC1: *p*-value < 0.0001, *R* = 0.51; PC2: *p*-value = 0.03, *R* = −0.29) and boys (PC1: *p*-value = 0.004, *R* = −0.40; PC2: *p*-value < 0.0001, *R* = −0.63).

To identify potential short-term exposure effects on gene expression, we analyzed the microarray data while using the mean PM_2.5_ exposure during the last month of pregnancy. We observed 432 (2.6%) genes of which the expression in boys and girls was differentially affected by exposure. For girls and boys, we identified 507 and 1144 genes respectively which were significantly associated with the last month of gestational PM_2.5_ exposure. Of these, there were 55 significant genes in overlap between boys and girls (See Additional file 1: Table S4). The top ten significant genes for each sex are given in Additional file 1: Table S5.

For boys, we found 180 genes significantly associated with both long- and short-term exposure, while 113 genes for girls.

Additional file 1: Figure S2C and D show the association of the first and second (third) principal component score with short-term PM_2.5_ exposure for girls and boys respectively. The first principal component was significantly associated with long-term PM_2.5_ exposure in both girls (PC1: *p*-value = 0.0005, *R* = 0.43; PC2: *p*-value = 0.20, *R* = 0.17) and boys (PC1: *p*-value < 0.0001, *R* = −0.58; PC2: *p*-value = 0.28, *R* = 0.16). For girls, the third principal component was significantly correlated with short-term PM_2.5_ exposure (PC3: *p*-value = 0.01, *R* = −0.31) and is therefore given on the y-axis in Additional file 1: Figure S2C.

Newborn sex-specific PM_2.5_ associated effects were further explored with overrepresentation analyses. The top 15 significant pathways with at least 15 measured genes and a total gene size of at most 500 genes are represented for both sexes in Tables 2 and 3 for long- and short-term PM_2.5_ exposure respectively. For each pathway, gene symbols and an indication of down- or up-regulation in association with PM_2.5_ exposure are given for the significant genes. For pathways with the same contributing genes, only the most significant pathway is shown.

**Table 2:** Top 15 overrepresented pathways associated with long-term PM_2.5_ exposure for girls and boys

Sex	Pathway	Effective/total size	# ↓ genes	Contributing genes	*P*-value
Girls					
	Generic Transcription Pathway^a^	367/478	80	Top 5 out of 33 significant genes: ZNF124↑; MED16↑; KRBA1↑; ZNF205↓; ZNF720↑	3.0E-06
	Defensins	19/53	15	ART1↓;DEFA3↓; DEFB1↓;DEFB128↑;DEFA4↓	5.7E-04
	Binding and Uptake of Ligands by Scavenger Receptors^a^	28/43	15	APOA1↓; HPR↓; HP↓; HBA2↑; FTL↑	3.6E-03
	agrin in postsynaptic differentiation	39/47	18	EGFR↑; PTK2↑; UTRN↑; ITGB1↑; CHRM1↓	1.5E-02
	JAK-STAT^a^	39/43	15	PTK2↑; ESR1↓; ZAP70↑;PDK1↑; ITGB1↑	1.5E-02
	ATM Signaling Pathway^a^	15/18	7	ATM↑;ATF2↑;RAD51↓	1.7E-02
	Integrated Pancreatic Cancer Pathway	141/165	62	SERPINB10↓;CAMP↓;RAD51↓;TYMS↓;INHBA↓; NTRK1↓;ATM↑;EGFR↑	1.8E-02
	Transcriptional misregulation in cancer - *Homo sapiens* (human)	146/179	73	CEBPE↓; CDKN2C↓; EWSR1↑; DEFA3↓; HIST1H3J↓; PTK2↑; ASPSCR1↓; MPO↓; NTRK1↓; ELANE↓; ATM↑	2.3E-02
	BARD1 signaling events	29/29	17	RAD51↓;ATM↑;EWSR1↑;UBE2D3↑	2.3E-02
	Gastric cancer network 2	29/32	9	CACYBP↑;AHCTF1↑;EGFR↑;BRIX1↑	2.3E-02
	Extracellular matrix organization	167/275	92	ITGB1↑; ELANE↓; MMP17↓; LTBP3↑; PLOD1↑; CTSG↓; CEACAM1↓; MMP8↓; CEACAM6↓; CEACAM8↓; COL17A1↓	2.5E-02
	Urokinase-type plasminogen activator (uPA) and uPAR-mediated signaling	32/45	15	CTSG↓;EGFR↑;ELANE↓;ITGB1↑	3.2E-02
	Downregulation of SMAD2/3:SMAD4 transcriptional activity	20/21	3	UBA52↑; TGIF2↑; PPM1A↑	3.8E-02
	JAK-STAT-Core	67/104	29	IL11RA↑; IL12RB1↑; STAT4↑; MPL↑; EGFR↑; IL6ST↑	4.1E-02
Boys					
	TNF receptor signaling pathway	44/48	29	IKBKB↑; MAP4K5↑; TAB2↑; TAB1↓; MAP2K3↑; TNIK↓; TNF↓; IKBKG↑; GNB2L1↓	4.8E-03
	Mercaptopurine Action Pathway	38/47	21	ATIC↓; PAICS↓; TPMT↓; APRT↓; ITPA↓; ADA↓; ABCC4↓; ADSL↓	6.5E-03
	Primary immunodeficiency - Homo sapiens (human)	32/36	25	ICOS↓; ORAI1↓; CD40LG↓; IKBKG↑; ADA↓; CD3D↓; LCK↓	8.6E-03
	the co-stimulatory signal during t-cell activation	18/21	13	CTLA4↓; ICOS↓; CD3D↓; LCK↓; ITK↓	9.0E-03
	FCERI mediated NF-kB activation^a^	19/63	13	IKBKB↑; TAB2↑; TAB1↓; RASGRP1↓; IKBKG↑	1.1E-02
	p73 transcription factor network	68/81	38	GNB2L1↓; PFDN5↑; PLPP1↓; UBE4B↑; TP73↓; BAK1↓; FOXO3↑; ADA↓; MDM2↑; BIN1↓; MYC↓	1.2E-02
	Axon guidance - Homo sapiens (human)^a^	88/127	57	EPHB2↓; EPHB3↓; ROBO2↓; PPP3R1↑; ROBO3↓; EFNA4↓; EFNA3↓; ROCK1↑; EPHA1↓; ITGB1↑; RGS3↓; ABLIM1↓; SEMA4C↓	1.4E-02
	Thiopurine Pathway, Pharmacokinetics/Pharmacodynamics	28/32	22	PRPS1↓; TPMT↓; NT5E↓; ITPA↓; ADA↓; ABCC4↑	1.6E-02
	T cell receptor signaling pathway - Homo sapiens (human)	91/104	59	DLG1↑; CTLA4↓; ICOS↓; RASGRP1↓; CD40LG↓; ITK↓; PPP3R1↑; IKBKB↑; TNF↓; CDK4↓; IKBKG↑; CD3D↓; LCK↓	1.8E-02
	Oncogene Induced Senescence^a^	29/31	13	TP53↓; E2F2↑; CDK4↓; TFDP1↑; MDM2↑; AGO3↑	1.9E-02
	Regulation of nuclear beta catenin signaling and target gene transcription	64/81	39	TCF7↓; HDAC2↓; TBL1XR1↑; AES↓; CAMK4↓; TNIK↓; APC↑; MYC↓; LEF1↓; AXIN2↓	2.1E-02
	TP53 Network	15/18	7	MDM2↑; TP53↓; MYC↓; TP73↓	2.2E-02
	Bladder Cancer	23/26	14	CDK4↓; TYMP↓; MDM2↑; TP53↓; MYC↓	2.6E-02
	Stimuli-sensing channels	68/102	38	TRPV6↓; CLCN3↑; WNK2↓; TRPM5↓; CLCN7↑; ANO10↓; TPCN1↑; BEST4↓; WWP1↓; WNK1↑	3.0E-02
	Amyotrophic lateral sclerosis (ALS) - Homo sapiens (human)	41/51	23	NEFH↓; PPP3R1↑; TOMM40↓; TNF↓; BCL2L1↑; MAP2K3↑; TP53↓	3.2E-02

# ↓ genes: number of down-regulated genes. ^a^Pathways that remain significant in the sensitivity analysis

**Table 3:** Top 15 overrepresented pathways associated with short-term PM_2.5_ exposure for girls and boys

Sex	Pathway	Effective /total size	# ↓ genes	Contributing genes	*P*-value
Girls					
	mRNA Processing^a^	124/126	32	PTBP2↑; SRSF1↑; SFPQ↑; SNRNP40↑; CELF1↑; HNRNPU↑; TRA2B↑; SRSF6↑; HNRNPH1↑; PRPF40A↑	1.9E-03
	Ephrin signaling	16/22	5	NCK2↑; SDCBP↑; ARHGEF7↑	8.4E-03
	Ectoderm Commitment Pathway^a^	87/129	30	PDE7A↑; SDCBP↑; MZF1↑; C1GALT1↑ NF2↑; OGT↑; TSC22D1↑	9.1E-03
	IL-4 Signaling Pathway^a^	52/53	27	IKBKB↑; PTPN11↑; IL2RG↓; ATF2↑; RPS6KB1↑	1.3E-02
	Physiological and Pathological Hypertrophy of the Heart^a^	20/24	8	IL6ST↑; CAMK2D↑; PPP3CB↑	1.6E-02
	miR-targeted genes in lymphocytes - TarBase	362/482	108	Top 5 out of 17 genes: MBNL1↑; SUCLG2↑; TGFBR2↑; GTPBP3↑; DMTF1↑	1.9E-02
	Basal transcription factors - Homo sapiens (human)^a^	39/45	13	TAF8↑; GTF2H2C_2↑; TAF1↑; TAF11↑	2.1E-02
	Spliceosome - Homo sapiens (human)^a^	127/130	33	HNRNPU↑; PRPF40A↑; RBM25↑; SNRNP40↑; THOC1↑; SRSF1↑; SRSF6↑; TRA2B↑	2.2E-02
	Activated TLR4 signaling^a^	110/120	47	ATF2↑; SIGIRR↑; IL6ST↑; PTPN11↑; IKBKB↑; IRF3↑; UBE2D3↑	2.9E-02
	Insulin Pathway	44/47	13	RPS6KB1↑; PTPN11↑; NCK2↑; EXOC7↑	3.0E-02
	Salmonella infection - Homo sapiens (human)	68/86	38	PFN1↓; RAB7A↓; DYNC1H1↑; WAS↓; PKN2↑	3.7E-02
	Amphetamine addiction - Homo sapiens (human)^a^	48/68	21	PPP3CB↑; CAMK2D↑; ATF4↑; ATF2↑	4.0E-02
	Generic Transcription Pathway	367/478	91	Top 5 out of 16 genes: ZNF625↑; ZNF37A↑; ZNF419↑; ZNF205↓; ZNF12↑	4.0E-02
	Direct p53 effectors^a^	123/147	47	PMS2↑; KAT2A↑; BNIP3L↑; TGFA↓; PIDD1↑; AIFM2↑; HIC1↓	4.9E-02
Boys					
	Lidocaine (Local Anaesthetic) Action Pathway^a^	19/31	13	CYP3A4↓; CACNA2D2↓; ATP1A4↑; ATP1B1↑; ATP1B3↑; ADRA1A↓	9.9E-04
	Signaling events mediated by PRL^a^	20/23	4	CDK2↑; BCAR1↓; RABGGTA↑; PTP4A3↑; ROCK1↑; ITGB1↑	1.3E-03
	Protein processing in endoplasmic reticulum - Homo sapiens (human)^a^	156/168	36	Top 5 out of 19 significant genes: ATF4↑; SEC31A↑; UBQLN3↓; UGGT1↑; CRYAB↓	6.2E-03
	Basigin interactions^a^	19/30	6	ATP1B3↑; SLC3A2↑; ATP1B1↑; CAV1↓; ITGB1↑	6.3E-03
	Morphine Action Pathway^a^	27/44	16	DNAJB11↑; CACNA2D2↓; ATP1A4↑; ATP1B1↑; ATP1B3↑; ADRA1A↓	6.9E-03
	mRNA Splicing - Major Pathway^a^	116/131	14	Top 5 out of 15 significant genes: SMC1A↑; PCBP1↑; PRPF8↑; SNRPA↓; CD2BP2↑	8.3E-03
	Validated transcriptional targets of AP1 family members Fra1 and Fra2	30/37	11	ATF4↑; TXLNG↑; LAMA3↓; NFATC3↑; USF2↑; CDKN2A↓	1.2E-02
	Maturity onset diabetes of the young - Homo sapiens (human)	15/25	14	NR5A2↓; PAX4↓; FOXA2↓; GCK↓	1.4E-02
	Hedgehog ligand biogenesis^a^	15/21	5	OS9↑; DISP2↓; P4HB↑; VCP↑	1.4E-02
	Salivary secretion - Homo sapiens (human)^a^	60/90	28	ADCY3↓; ADRA1A↓; NOS1↓; GUCY1A3↑; PRH2↓; ATP1A4↑; ATP1B1↑; ATP1B3↑; ATP2B3↓	1.5E-02
	Processing of Capped Intron-Containing Pre-mRNA^a^	147/162	18	Top 5 out of 17 significant genes: SMC1A↑; RANBP2↑; PCBP1↑; PRPF8↑; SNRPA↓	1.5E-02
	G. alpha (s) signaling events^a^	81/129	44	ADCYAP1R1↓; CALCA↓; PTH2↓; ADCY3↓; GNAZ↓; TSHB↓; INSL3↓; TAAR2↓; GHRHR↓; GLP2R↓; GNG13↓	1.6E-02
	Neuroactive ligand-receptor interaction - Homo sapiens (human)	164/275	102	GABRG2↓; GABRP↓; NTSR2↓; TAAR2↓; TSHB↓; CHRM5↓; ADCYAP1R1↓; GH1↓; GHRHR↓; HTR1B↓; ADRA1A↓; GLP2R↓; THRA↓; ADORA1↓; CHRNA2↓; LPAR1↑; OPRL1↓; GRM5↓	2.1E-02
	FOXM1 transcription factor network	34/42	10	CDK2↑; XRCC1↑; CENPF↑; NFATC3↑; TGFA↓; CDKN2A↓	2.1E-02
	TCA Cycle	17/17	4	FH↑; MDH2↑; OGDH↑; IDH2↑	2.2E-02

# ↓ genes: number of down-regulated genes. ^a^Pathways that remain significant in the sensitivity analysis

For girls, “Generic Transcription Pathway” and “Defensins” were the top most significant pathways in relation to long-term PM_2.5_ exposure including 22% and 79% down-regulated genes respectively (Table 2). Both α- and β-defensins, involved in host defense and chronic inflammatory responses, were deregulated by long-term PM_2.5_ exposure. Among the 11 measured genes specifically encoding defensin peptides, 9 were down-regulated. Other significant pathways were related to DNA damage response, cancer, signaling transduction, scavenging, and the extracellular matrix.

For boys, the “Tumor necrosis factor (TNF) receptor signaling pathway” was most significantly associated with long-term PM_2.5_ exposure (Table 2). Other top significant pathways were mostly involved in the immune response or were related to cancer or the nervous system. Long-term PM_2.5_ was associated with lower expression of various genes of the ephrin family [e.g. ephrins (*EPH*) and EPH-related receptors (*EFN*)] and members of the Roundabout (ROBO) family [e.g. *ROBO2* and *ROBO3*].

For the pathways “Oncogene Induced Senescence”, “TP53 Network”, and “Bladder Cancer”, we observed a down-regulation of tumor protein p53 (*TP53*) and an increase of Mouse double minute 2 homolog (*MDM2*) expression, an important inhibitor of TP53 transcriptional activation.

For girls, overrepresentation analysis for short-term PM_2.5_ exposure revealed pathways related to transcriptional regulation, immune response, embryonic development, cardiovascular system, and response to DNA damage (Table 3).

For boys, the top significant pathway for short-term PM_2.5_ exposure was “Lidocaine (Local Anaesthetic) Action Pathway” which contains gene encoding voltage-gated sodium channels in peripheral neurons (Table 3). Other significant pathways were “Hedgehog ligand biogenesis” important for embryonic development, “Tricarboxylic acid (TCA) cycle” responsible for energy production, and “Neuroactive ligand-receptor interaction - Homo sapiens (human)” including several neurotransmitter receptor encoding genes which are negatively associated with short-term PM_2.5_ exposure.

Clusters of functional related pathways, modulated by long- and short-term PM_2.5_ exposure, are presented in Additional file 1: Figure S3 and S4 respectively. Each cluster is encircled and assigned a label. Tables 4 and 5 list the cluster labels and the corresponding individual pathways which were significantly up- or downregulated by long- and short-term PM_2.5_ exposure respectively. Table 4 shows the GSEA results for long-term exposure in girls which were consistent with the overrepresentation analysis for 1) the pathways “Defensins” and “Extracellular matrix organization”, which both were down-regulated, and for 2) the pathways related to Transcription-SMAD2, 3, 4-TGFβ which were up-regulated. Additional pathways were related to the cell cycle (“FOXM1” and “Aurora B pathway”) and pathways containing genes encoding histone peptides, ribosomal peptides, and olfactory receptors.

**Table 4:** Pathways modulated by long-term PM_2.5_ exposure for girls and boys resulting from GSEA

Sex	Cluster label	Source: pathway	# genes	Direction of regulation
Girls	Aurora B pathway	PID: Aurora B pathway	36	DOWN
	Core matrisome	Matrisome: Naba core matrisome	142	DOWN
	Defensins	Reactome: defensins	18	DOWN
	Extracellular matrix organization			DOWN
		Reactome: extracellular matrix organization	49	
		Reactome: degradation of the extracellular matrix	18	
	FOXM1 pathway	PID: FOXM1 pathway	32	DOWN
	Histone related pathways			DOWN
		Reactome: amyloids	69	DOWN
		Reactome: RNA polymerase I promotor opening	54	DOWN
		KEGG: systemic lupus erythematosus	116	DOWN
	Olfactory signaling			DOWN
		KEGG: olfactory transduction	124	DOWN
		Reactome: olfactory signaling pathway	95	DOWN
	Porphyrin metabolism	KEGG: porphyrin and chlorophyll metabolism	25	DOWN
	Ribosome related pathways			UP
		Reactome: peptide chain elongation	83	
		KEGG: ribosome	85	
		Reactome: nonsense mediated decay enhanced by the exon junction complex	104	
	Transcription-SMAD2,3,4-TGFβ pathways			UP
		Reactome: generic transcription pathway	267	
		Reactome: downregulation of SMAD2, 3, SMAD4 transcriptional activity	18	
		Reactome: signaling by TGF-beta receptor complex	54	
Boys	Apoptotic execution	Reactome: apoptotic execution phase	43	UP
	Cell cycle			UP
		Reactome: cell cycle mitotic	290	
		Reactome: mitotic prometaphase	79	
		Reactome: DNA replication	176	
	HDAC class III	PID: HDAC class III pathway	22	UP
	UPA-UPAR pathway	PID: uPA uPAR pathway	30	UP
	RhoA pathway	PID: RhoA pathway	37	UP

For clusters containing more than 3 pathways, only the top 3 significant pathways are given.

# gene: number of genes within a pathway. *uPAR* Urokinase-type plasminogen activator (uPA) receptor, *HDAC* histone deacetylase, *FOXM1* forkhead box M1, *RhoA* Ras homolog gene family member A, *PID* Pathway Interaction Database, *KEGG* Kyoto Encyclopedia of Genes and Genomes

**Table 5:** Pathways modulated by short-term PM_2.5_ exposure for girls and boys resulting from GSEA

Sex	Cluster label	Source: pathway	# genes	Direction of regulation
Girls	Olfactory signaling	Reactome: olfactory signaling pathway	95	DOWN
	Rho pathway	BioCarta: Rho pathway	28	DOWN
	Ribosome related pathways			UP
		Reactome: nonsense mediated decay enhanced by the exon junction complex	104	
		KEGG: ribosome	85	
		Reactome: 3′ UTR mediated translational regulation	102	
Boys	ATM pathway	PID: ATM pathway	18	UP
	BARD1 pathway	PID: BARD1 pathway	29	UP
	Cell Cycle			UP
		Reactome: DNA replication	176	
		Reactome: G2/M checkpoints	37	
		Reactome: cell cycle mitotic	290	
	ETC-TCA cycle			UP
		Reactome: TCA cycle and respiratory electron transport	113	
		Reactome: respiratory electron transport atp synthesis by chemiosmotic coupling and heat production by uncoupling proteins	79	
	M-calpain pathway	BioCarta: M-calpain pathway	21	UP
	Metabolism of mRNA and RNA			UP
		Reactome: metabolism of RNA	249	
		Reactome: metabolism of mRNA	204	
	Myc pathway	PID: Myc activ pathway	76	UP
	Olfactory signaling			DOWN
		KEGG: olfactory transduction	124	
		Reactome: olfactory signaling pathway	95	
	mRNA processing			UP
		Reactome: processing of capped intron containing pre mRNA	133	UP
		Reactome: mRNA processing	147	
	Response to elevated platelet cytosolic CA^2+^	Reactome: response to elevated platelet cytosolic CA^2+^	66	UP
	Ribosome related pathways			UP
		Reactome: translation	141	
		Reactome: SRP dependent cotranslational protein targeting to membrane	105	
		Reactome: 3′ UTR mediated translational regulation	102	
	RhoA pathway	PID: RhoA pathway	37	UP
	Splicesome	KEGG: spliceosome	123	UP

For clusters containing more than 3 pathways, only the top 3 significant pathways are given

# genes: number of genes within a pathway. *Rho* Ras Homolog gene family, *TCA* tricarboxylic acid, *ETC* electron transport chain, *ATM* Ataxia Telangiectasia Mutated, *BARD1* BRCA1 associated RING domain 1. *Myc* v-myc avian myelocytomatosis viral oncogene homolog, *PID* Pathway Interaction Database, *KEGG* Kyoto Encyclopedia of Genes and Genomes

For boys, the top significant pathways modulated by long-term PM_2.5_ exposure were all up-regulated (Table 4) and were related with cell cycle, plasminogen activation system (UPA-UPAR pathway), execution phase of apoptosis, Ras homolog gene family member A (RhoA) pathway, and regulation of gene expression by histone deacetylase (HDAC) class III. The 18 “leading edge genes” of the RhoA pathway included among others Diaphanous-Related Formin 1 (*DIAPH1*), Rho-Associated Coiled-Coil Containing Protein Kinase 1 (*ROCK1*), and *ROCK2* of which the gene products are effectors of RhoA. Two of these effectors, *ROCK1* and *DIAPH1* were significantly associated with long-term PM_2.5_ exposure. Plasminogen activation system was also PM_2.5_ sensitive in girls (Table 2).

For girls, GSEA results for short-term PM_2.5_ exposure revealed significantly up-regulated pathways related to ribosomes and significantly down-regulated pathways related to the Rho pathway and olfactory signaling (Table 5). As found before in girls for long-term exposure, both olfactory signaling and ribosome related pathways were also significantly associated with short-term PM_2.5_exposure. The Rho pathway contained 12 “leading edge genes” including *RHOA*, *DIAPH1*, LIM domain kinase 1 (*LIMK1*), Cofilin 1 (*CFL1*), several members of the Rho guanine nucleotide exchange factors (ARHGEF) family, and genes encoding subunits of the Actin Related Protein 2/3 Complex. However, none of these genes were significantly associated with short-term PM_2.5_ exposure.

For boys, there were 132 significantly up-regulated and 11 down-regulated pathways by short-term PM_2.5_ exposure. Because of the large amount of significant pathways, Table 5 represents only the pathways with both *p*-value and q-value smaller than 0.005. Most of the significant pathways were up-regulated and linked to the cell cycle or ribosomes. Other up-regulated pathways were related to the TCA cycle and DNA damage response including “BRCA1 Associated RING Domain 1 (BARD1) pathway” and “Ataxia Telangiectasia Mutated (ATM) pathway”. The 23 “leading edge genes” of the BARD1 pathway included among others *BARD1*, Breast Cancer 1 Early Onset (*BRCA1*), and *ATM*. Note that “BARD1 pathway” and “ATM pathway” were also significantly associated with long-term PM_2.5_ exposure in girls (Table 2). The RhoA pathway results were similar as those for long-term PM_2.5_ exposure. *DIAPH1* and *ROCK1* were both significantly associated with short-term PM_2.5_ exposure and contributed to the “leading edge genes”. Down-regulated pathways were related to olfactory receptor signaling pathways.

It has been reported that air pollution exposure can induce changes in WBC counts in adults [35, 36], and changes in cord blood cell distribution might influence the overall blood transcriptome profile. However, in our newborn cohort, we did not find a significant association between PM_2.5_ exposure and WBC count and neutrophil percentage in cord blood. Nevertheless, in a sensitivity analysis we added WBC count and neutrophil percentage to the main model. For girls, 525 (72.5%) of the significant genes in the main analysis remained significantly associated with long-term PM_2.5_ exposure after adjustment for WBC count and neutrophil percentage. Overrepresented pathways of the main analysis that remained significant in the sensitivity analysis are marked (^a^) (Table 2). For GSEA, pathways related to defensins, histones (“Amyloids”), extracellular matrix organization, and olfactory receptors remained in the top most significant pathways.

For boys, 773 (56.9%) of the significant genes associated with long-term PM_2.5_ exposure in the main analysis remained significant after adjustment for WBC count and neutrophil percentage. GSEA confirmed our main findings with pathways related to the cell cycle (q-value <0.25 and *p*-value <0.005) including “Mitotic M-M/G1 phases”, “Cell cycle mitotic”, and “Loss of Ninein-Like Protein (NLP) from mitotic centrosomes”. For girls, 433 (85.4%) genes which significantly correlated with short-term PM_2.5_ exposure in the main analysis were in overlap with the sensitivity analysis. Of the top 15 significant enriched pathways for short-term PM_2.5_ exposure in girls (Table 3), nine pathways remained significantly overrepresented in the sensitivity analysis. No significant up-regulated pathways resulted from GSEA, however, ribosome related pathways had the most significant positive association with short-term PM_2.5_ exposure. Pathways related to olfactory signaling remained significantly down-regulated.

For boys, 1055 (92.2%) of the significant genes in the main analysis remained significantly correlated with short-term PM_2.5_ exposure in the sensitivity analysis. The most significant overrepresented pathway after adjustment for blood count was proteasome complex of which all ten contributing genes were up-regulated. Eight of these genes encoded proteasome subunits. Of the top 15 significant pathways in the main overrepresentation analysis, ten pathways remained significantly enriched in the sensitivity analysis (Table 3). GSEA revealed 134 significantly up-regulated and 13 down-regulated pathways. All pathways shown in Table 5 remained significant except the “M-calpain pathway”.

## Discussion

This is the first paper reporting neonate transcriptome signatures for long-term and short-term gestational exposure to PM. Although epidemiological studies are scarce, transcriptome alterations in early life may act in response to environmental exposures heralding adverse health outcomes later in life. At the gene level we observed in cord blood substantial differences in transcriptomic responses between newborn girls and boys in association with air pollution exposure during pregnancy. However, pathway analyses revealed alterations in the immune and DNA damage responses in both sexes for long-term exposure. Considering short-term exposure (last month of pregnancy), significant pathways were identified for both girls and boys which were related to olfactory receptors, ribosomes, and DNA damage. For long-term exposure, we also found sex-specific pathways including “axon guidance” and “RhoA pathway” for boys, while olfactory receptor, cell cycle, ribosomal, and defensin-related processes were girl-specific. Sex-specific pathways associated with short-term exposure in boys included processes involved in synaptic transmission (“neuroactive ligand-receptor interaction”) and mitochondrial energy production, and for girls immune response pathways. Table 6 gives an overview of these biological processes altered by gestational PM exposure.

**Table 6:**
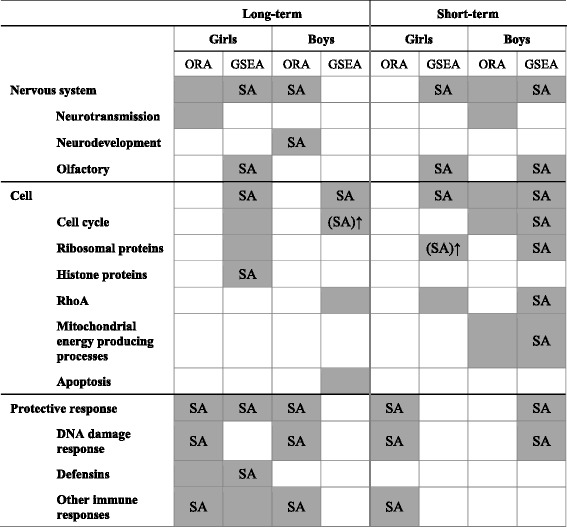
Overview of selected biological processes altered by gestational PM exposure

*ORA* Overrepresentation Analysis. *GSEA* Gene Set Enrichment Analysis

Gray: PM_2.5_-related processes in the main analysis. SA: processes that remained significant in the sensitivity analysis. (SA)↑: most significant up-regulated pathways in the sensitivity analysis

We suggest that the observed inverse association between gene expression of olfactory receptors could be an early marker of the effects of fine particle air pollution on the central nervous system. An association between air pollution exposure and olfactory dysfunction has been suggested to be involved in the development of various diseases such as Alzheimer and Parkinson’s disease [37]. Importantly, the functional role of gene expression of olfactory receptors in blood parallels severity of head injury as indicated in patients suffering of traumatic brain injury [38].

Besides olfactory receptor signaling, we identified other neurological pathways affected by long- and short-term PM_2.5_ exposure in boys. Long-term exposure down-regulated the expression of ROBO, EPH and EFN members which are essential for axon guidance during neurodevelopment. Short-term PM_2.5_ exposure altered expression of “Neuroactive ligand-receptor interaction - Homo sapiens (human)” gene members including several types of neurotransmitter receptor encoding genes such as gamma-aminobutyric acid (GABA) receptors, cholinergic and glutamate receptors. Interestingly, all these contributing genes were negatively correlated with PM_2.5_ exposure. In mice, decreased expression of ionotropic glutamate receptor subunit in the hippocampus of offspring was shown following gestational exposure to benzo(a)pyrene [39]. In rats, exposure to cigarette smoke showed a dose-dependent decrease of GABA B receptor, 1 mRNA expression in the hippocampus [40]. Changes in neurotransmitter receptor expressions early in life are predictive for cognitive dysfunction and behavior deficits later in life [41].

In adults, the increased risk in lung cancer associated with ambient air pollution is suspected to be linked to genotoxic chemicals absorbed on PM, more specifically polycyclic aromatic hydrocarbons (PAH) [42], and toxic metals e.g. cadmium [43]. Fetuses are more susceptible to carcinogenic exposures due to their rapid cell proliferation and differentiation, greater absorption and retention, immature immune system, and decreased capacity of detoxification, DNA repair or apoptotic [44, 45]. Micronuclei, a validated biomarker of cancer risk, are extranuclear bodies originating from dividing cells that are formed by chromosomal breakage and/or whole chromosome loss [46]. A Danish birth cohort showed that micronuclei frequencies, measured in cord blood, were elevated among newborns whose mothers lived in high-traffic-density areas [47]. In our study, we identified several pathways that may underlie the carcinogenic potential of air pollution in early life. “ATM” and “BARD1” pathways were significantly modulated by PM_2.5_ exposure for short-term exposure in boys and long-term exposure in girls. These pathways play a central role in the response to DNA damage and may be important in the potential of PM_2.5_ to induce genotoxic stress. Jiang et al. found elevated *ATM* expression in esophageal squamous cell carcinoma specimens of smokers compared to non-smokers [48].

Other pathways related to DNA damage which were significantly associated with long-term PM_2.5_ exposure were “p73 transcription factor network”, “Oncogen induced Senescence”, and “*TP53* network” in boys only. At the gene level the up-regulation of *MDM2*, a negative regulator of *TP53*, is in line with the inverse association of long-term PM_2.5_ exposure and *TP53* expression and its family member *TP73*. In contrast to our observations, Rossner et al. reported positive associations between p53 protein plasma levels and personal PAH exposure in city policemen and bus drivers at work [49].

Expression of these DNA damage responsive genes seem to be affected by PM_2.5_ exposure in a time dependent manner. It is plausible that deregulated gene expression of key players of the response to DNA damage, as a consequence of fine particle air pollution exposure, may increase the susceptibility to develop cancer and other diseases later in life.

The positive association between expression of gene members of the RhoA pathway, which are important for cytoskeleton organization, and gestational long- and short-term PM_2.5_ exposure for boys supports the idea that air pollution can activate the Rho/ROCK pathway [50, 51] potentially through increased production of reactive oxygen species (ROS) [52]. Our findings are consistent with those of Sun et al. who found increased expression levels of *ROCK1* but not *ROCK2* and *RhoA,* in aortic tissue of PM_2.5_-exposed rats compared with rats exposed to filtered air after they were infused with angiotensin II [51]. Along similar lines, evidence in aorta of mice indicated that the RhoA/ROCK pathway plays a fundamental role in PM_2.5_-mediated myocardial remodeling and hypertension [53].

Sex-specific pathways included “defensins” for girls. Most of the genes encoding defensin peptides were down-regulated with increasing long-term PM_2.5_ exposure. Defensins are host defense peptides with antibacterial activity and represent major components of innate immunity. Two subfamilies of defensins, α- and β-defensins, are present in humans: α-defensins are mainly stored in granules of neutrophils and intestinal Paneth cells, while β-defensins are expressed in various epithelial cells. Interestingly, the gene expression of elastase (*ELANE*) and cathepsin G (*CTSG*, proteases interacting with precursors of α-defensins [54]), were in the current study also significantly down-regulated and are members of the overrepresented “Urokinase-type plasminogen activator (uPA)” and “uPAR-mediated signaling pathway” (Table 2). Previous studies found a negative association between β-defensin gene expression and residential fly ash, one of the residues generated by oil combustion and being a potential component of PM_2.5_ [55, 56]. Decreased levels of antimicrobial peptides, including defensins, may result in higher susceptibility to infections as observed in preterm neonates [57, 58].

For boys, several immune response pathways involved in both TNF-NF-KB (nuclear factor of kappa light polypeptide gene enhancer in B-cells) and T cell receptor signaling were associated with long-term PM_2.5_ exposure. After adjustment for blood cell count these pathways were no longer significant.

Mitochondria, the energy producers of the cells, are particularly sensitive to environmental toxicants due to their lack of DNA repair capacity. Fetuses may adapt their mitochondrial structure and function when the supply of nutrients is limited. Previously, we showed in the ENVIR*ON*AGE birth cohort that placental mitochondrial DNA content [21] and epigenetic modifications [59] in the mitochondrial genome were associated with PM exposure during pregnancy. In line with these findings, we revealed that mitochondrial tricarboxylic acid cycle and respiratory electron chain pathways were significantly linked to short-term gestational PM_2.5_exposure in boys.

The advantage of our study is that we used a standardized fine-scale exposure assessment enabling us to calculate both short- and long-term exposure on a high resolution scale. Exposure levels in our study were comparable with other European cohort studies. Our study has limitations. First, observational studies do not allow to establish causality. Second, the observed gene expression changes in umbilical cord blood are only indirect evidence of the effects on fetal target tissues such as cardiovascular and nervous tissue. We identified several PM_2.5_-altered genes involved in neural development. A review of 18 studies [60] evaluating comparability of peripheral blood and brain transcriptome data in adults estimated cross-tissue correlation between 0.25 and 0.64 with stronger associations for some subsets of genes and biological processes. Novartis human transcriptomic data [61] showed the following median correlation coefficients between gene expression in whole blood and tissues: immune tissues (*R* = 0.64), central nervous system (*R* = 0.50), peripheral nervous system (*R* = 0.36), heart (*R* = 0.48), and fetal brain (*R* = 0.54). These results support to some extent the use of peripheral blood transcriptome data as surrogate for gene expression in other tissues such as the central nervous system [60, 61]. Maron et al. [62] identified fetal biomarkers by comparing gene expression profiles from both maternal and umbilical cord blood in humans. Interestingly, several of the identified transcripts present in both maternal and fetal circulation were identified to be affected by PM_2.5_ exposure in our study both in gene and pathway analysis. This includes immunological and olfactory receptor gene transcripts as well as genes important for development of the nervous system (see Tables 2 and 3 and Maron et al. [62]). Third, our study included 26 (18%) smokers. We adjusted our analyses for maternal smoking status. Although smoking is a major source of personal air pollution exposure, it is unlikely that this biased the current results as we did not find a significant association between maternal smoking and residential air pollution levels. Lastly, the long-term PM_2.5_ concentration in our study ranged from 11.8 to 20.6 μg/m^3^, with an interquartile range of 2.34 μg/m^3^. Although this exposure contrast is relatively narrow, previously even smaller contrasts in exposure has been reported in epidemiological studies studying hard clinical endpoints, e.g. the Worcester Heart Attack Study [63] reported a link with acute myocardial infarction for an interquartile range PM_2.5_ exposure contrast of 0.59 μg/m^3^. Nevertheless, we acknowledge that the small range of PM_2.5_ exposure and the large number of tests in combination with a small sample size reduces the power of our study. In this regard, we did not apply false discovery rate correction on the individual genes. To improve the reliability of our results, we focused on significant pathways and their genes instead of individual genes. We applied two approaches for the pathway analysis to fully understand the impact of prenatal PM_2.5_ exposure on gene expression: ORA which is based on the *p*-value of individual genes and GSEA which uses the fold change to identify significant pathways. GSEA does not require the use of a significance cut-off at gene level, thereby overcoming the issue of multiple testing. Although the low power of the current study due to the small range of PM_2.5_ exposure in the study region, we believe our study can serve as an exploratory analysis which may inspire further research in this area.

## Conclusions

To our knowledge, this is the first study showing a sex-specific link between gestational fine particles and whole genome gene expression in cord blood. The identified transcriptome pathways could provide new molecular insights as to the interaction pattern of early life PM_2.5_ exposure with the biological development of the fetus.

## Additional file

Additional file 1: Table S1. Descriptive characteristics of the ENVIR*ON*AGE birth cohort participants compared to all births in Flanders (Northern part of Belgium) from 2002 to 2011. **Table S2.** Significant differentially expressed genes by long-term PM_2.5_ exposure in cord blood of girls and boys. **Table S3.** Top ten significant genes in cord blood of newborn boys and girls associated with long-term PM_2.5_ exposure. **Table S4.** Significant differentially expressed genes by short-term PM_2.5_ exposure in cord blood of girls and boys. **Table S5.** Top ten significant genes in cord blood of newborn boys and girls associated with short-term PM_2.5_ exposure. **Figure S1.** Histogram representing the percentage of genes with *p*-value <0.05 for each variable included in the model. **Figure S2.** Principal component analysis plot showing the transcriptomic response to long- and short-term PM_2.5_ exposure in *(A, C)* girls and *(B, D)* boys. **Figure S3.** Pathways modulated by long-term PM_2.5_exposure for girls (*A*) and boys (*B*) resulting from GSEA. **Figure S4.** Pathways modulated by short-term PM_2.5_ exposure for girls (*A*) and boys (*B*) resulting from GSEA. (DOCX 1749 kb)

## Abbreviations

BMI: body mass index
Environage: ENVIRonmental influence ON early AGEing
FDR: false discovery rate
GO: gene ontology
GSEA: Gene Set Enrichment Analysis
IFDM: Immission Frequency Distribution Model
ORA: overrepresentation analysis
PAH: polycyclic aromatic hydrocarbons
PM: particulate matter
PM_2.5_: particulate matter with a diameter < 2.5 μm
R: partial correlation coefficients
ROS: reactive oxygen species
SA: sensitivity analysis
SD: standard deviation
TCA: tricarboxylic acid
WBC: white blood cell
