# Supplementary material for: Newborn sex-specific transcriptome signatures and gestational exposure to fine particles: findings from the ENVIRONAGE birth cohort
Source: Environ Health. 2017 Jun 5;16:52. doi: 10.1186/s12940-017-0264-y (PMC5458481; doi:10.1186/s12940-017-0264-y)
Supplement: Additional file 1: Table S1. — Descriptive characteristics of the ENVIRONAGE birth cohort participants compared to all births in Flanders (Northern part of Belgium) from 2002 to 2011. Table S2. Significant differentially expressed genes by long-term PM2.5 exposure in cord blood of girls and boys. Table S3. Top ten significant genes in cord blood of newborn boys and girls associated with long-term PM2.5 exposure. Table S4. Significant differentially expressed genes by short-term PM2.5 exposure in cord blood of girls and boys. Table S5. Top ten significant genes in cord blood of newborn boys and girls associated with short-term PM2.5 exposure. Figure S1. Histogram representing the percentage of genes with p-value <0.05 for each variable included in the model. Figure S2. Principal component analysis plot showing the transcriptomic response to long- and short-term PM2.5 exposure in (A, C) girls and (B, D) boys. Figure S3. Pathways modulated by long-term PM2.5exposure for girls (A) and boys (B) resulting from GSEA. Figure S4. Pathways modulated by short-term PM2.5 exposure for girls (A) and boys (B) resulting from GSEA. (DOCX 1749 kb) [file 12940_2017_264_MOESM1_ESM.docx]

# Additional file

**Newborn Sex-specific Transcriptome Signatures and Gestational Exposure to Fine Particles: Findings from the ENVIR*ON*AGE Birth Cohort**

Ellen Winckelmans_,_ Karen Vrijens, Maria Tsamou, Bram G. Janssen, Nelly D. Saenen, Harry A. Roels, Jos Kleinjans, Wouter Lefebvre, Charlotte Vanpoucke, Theo M. de Kok, Tim S. Nawrot

**Table of contents**

# Tables

# Table S1. Descriptive characteristics of the ENVIR*ON*AGE birth cohort participants compared to all births in Flanders (Northern part of Belgium) from 2002-2011.

# Table S2. Significant differentially expressed genes by long-term PM_2.5_ exposure in cord blood of girls and boys.

Table S3. Top ten significant genes in cord blood of newborn boys and girls associated with long-term PM_2.5_ exposure.

# Table S4. Significant differentially expressed genes by short-term PM_2.5_ exposure in cord blood of girls and boys.

Table S5. Top ten significant genes in cord blood of newborn boys and girls associated with short-term PM_2.5_ exposure.

# Figures

# Figure S1. Histogram representing the percentage of genes with p-value <0.05 for each variable included in the model.

# Figure S2. Principal component analysis plot showing the transcriptomic response to long- and short-term PM_2.5_ exposure in *(A, C)* girls and *(B, D)* boys.

# Figure S3. Pathways modulated by long-term PM_2.5_ exposure for girls (*A*) and boys (*B*) resulting from GSEA.

# Figure S4. Pathways modulated by short-term PM_2.5_ exposure for girls (*A*) and boys (*B*) resulting from GSEA.

# References

# Tables

# Table S1. Descriptive characteristics of the ENVIR*ON*AGE birth cohort participants compared to all births in Flanders (Northern part of Belgium) from 2002-2011.

| **Characteristic** | **ENVIR*ON*AGE subpopulation** **(n=142)** | **ENVIR*ON*AGE birth cohort**  **(n=673)^*^** | **Flanders§ (n=606,877)** |
| --- | --- | --- | --- |
| **Maternal** |  |  |  |
| Age, yrs | 29.4 (24.0-34.0) | 29.1 (23.0-35.0) | 29.5 (23.5-35.8) |
| Education |  |  |  |
| Low | 10.6 | 11.7 | 13.1 |
| Medium | 35.2 | 36.0 | 40.8 |
| High | 54.2 | 52.3 | 46.1 |
| Parity |  |  |  |
| 1 | 50.0 | 55.9 | 46.9 |
| 2 | 40.9 | 34.0 | 34.7 |
| ≥3 | 9.2 | 10.1 | 18.4 |
| **Newborn** |  |  |  |
| Sex |  |  |  |
| Boys | 45.8 | 49.8 | 51.4 |
| Ethnicity |  |  |  |
| European-Caucasian | 87.3 | 88.2 | 87.7 |
| Birth weight, g | 3457 (2910, 4045) | 3419 (2850, 4004) | 3360 (2740, 3965) |

Values are percentages or means (10^th^, 90^th^ percentiles). ^*^from 2010-2014. §([Cox et al. 2013](#_ENREF_1))

# Table S2. Significant differentially expressed genes by long-term PM_2.5_ exposure in cord blood of girls and boys.

| **Gene symbol** | **Gene name** |
| --- | --- |
| **Up-regulated genes for both sexes** | |
| AP3D1 | adaptor-related protein complex 3, delta 1 subunit |
| C1GALT1 | c core 1 synthase, glycoprotein-N-acetylgalactosamine 3-beta-galactosyltransferase 1 |
| CEP131 | centrosomal protein 131kDa |
| FBXW8 | F-box and WD repeat domain containing 8 |
| GTF2I | general transcription factor IIi |
| HECA | hdc homolog, cell cycle regulator |
| ITGB1 | integrin, beta 1 (fibronectin receptor, beta polypeptide, antigen CD29) |
| MAST3 | microtubule associated serine/threonine kinase 3 |
| NEAT1 | nuclear paraspeckle assembly transcript 1 (non-protein coding) |
| NOL10 | nucleolar protein 10 |
| RBM6 | RNA binding motif protein 6 |
| TBC1D31 | TBC1 domain family, member 31 |
| TBL1XR1 | transducin (beta)-like 1 X-linked receptor 1 |
| TPCN1 | two pore segment channel 1 |
| UBXN2A | UBX domain protein 2A |
| UHMK1 | U2AF homology motif (UHM) kinase 1 |
| UPF2 | UPF2 regulator of nonsense transcripts homolog (yeast) |
| UTRN | utrophin |
| ZFC3H1 | zinc finger, C3H1-type containing |
| **Down-regulated genes for both sexes** | |
| ART1 | ADP-ribosyltransferase 1 |
| ATG4B | autophagy related 4B, cysteine peptidase |
| BHLHE23 | basic helix-loop-helix family, member e23 |
| CACNA2D1 | calcium channel, voltage-dependent, alpha 2/delta subunit 1 |
| CACTIN | cactin, spliceosome C complex subunit |
| CDC42EP5 | CDC42 effector protein (Rho GTPase binding) 5 |
| CHRM1 | cholinergic receptor, muscarinic 1 |
| CYGB | cytoglobin |
| ESR1 | estrogen receptor 1 |
| HMG20B | high mobility group 20B |
| IP6K1 | inositol hexakisphosphate kinase 1 |
| KCTD19 | potassium channel tetramerization domain containing 19 |
| KRTAP2-4 | keratin associated protein 2-4 |
| LINC00320 | long intergenic non-protein coding RNA 320 |
| LINC00544 | long intergenic non-protein coding RNA 544 |
| MAP1S | microtubule-associated protein 1S |
| PRR25 | proline rich 25 |
| PRR36 | proline rich 36 |
| SCGB2B2 | secretoglobin, family 2B, member 2 |
| SNORD25 | small nucleolar RNA, C/D box 25 |
| SORBS3 | sorbin and SH3 domain containing 3 |
| TMEM151B | transmembrane protein 151B |
| **Up-regulated genes for boys, down-regulated for girls** | |
| CEACAM7 | carcinoembryonic antigen-related cell adhesion molecule 7 |
| DNAH10 | dynein, axonemal, heavy chain 10 |
| **Down-regulated genes for boys, up-regulated for girls** | |
| ALKBH2 | alkB homolog 2, alpha-ketoglutarate-dependent dioxygenase |
| APBA3 | amyloid beta (A4) precursor protein-binding, family A, member 3 |
| BCL11B | B-cell CLL/lymphoma 11B (zinc finger protein) |
| BEX2 | brain expressed X-linked 2 |
| CD248 | CD248 molecule, endosialin |
| CD6 | CD6 molecule |
| CECR5-AS1 | CECR5 antisense RNA 1 |
| CHCHD6 | coiled-coil-helix-coiled-coil-helix domain containing 6 |
| CIAO1 | cytosolic iron-sulfur assembly component 1 |
| DBH-AS1 | DBH antisense RNA 1 |
| EVL | Enah/Vasp-like |
| FARS2 | phenylalanyl-tRNA synthetase 2, mitochondrial |
| FBXO32 | F-box protein 32 |
| IL32 | interleukin 32 |
| MCF2L-AS1 | MCF2L antisense RNA 1 |
| PCED1B | PC-esterase domain containing 1B |
| PLAG1 | pleiomorphic adenoma gene 1 |
| PRPF39 | pre-mRNA processing factor 39 |
| RAB11FIP3 | RAB11 family interacting protein 3 (class II) |
| RASA4 | RAS p21 protein activator 4 |
| RPUSD3 | RNA pseudouridylate synthase domain containing 3 |
| SEMA4C | sema domain, immunoglobulin domain (Ig), transmembrane domain (TM) and short cytoplasmic domain, (semaphorin) 4C |
| TESPA1 | thymocyte expressed, positive selection associated 1 |
| THEMIS | thymocyte selection associated |
| TLDC1 | TBC/LysM-associated domain containing 1 |
| UBQLNL | ubiquilin-like |
| URB2 | URB2 ribosome biogenesis 2 homolog (S. cerevisiae) |
| WFS1 | Wolfram syndrome 1 (wolframin) |
| ZNF32 | zinc finger protein 32 |
| ZNF500 | zinc finger protein 500 |

Table S3. Top ten significant genes in cord blood of newborn boys and girls associated with long-term PM_2.5_ exposure.

| **Sex** | **Gene symbol** | **Gene name** | **FC** |
| --- | --- | --- | --- |
| **Girls (p-value <0.0025)** | | | |
|  | CDC42EP5 | CDC42 effector protein (Rho GTPase binding) 5 | 0.85 |
|  | ZNF404 | zinc finger protein 404 | 1.60 |
|  | SLC25A19 | solute carrier family 25 (mitochondrial thiamine pyrophosphate carrier), member 19 | 0.64 |
|  | SMYD3 | SET and MYND domain containing 3 | 1.64 |
|  | THEM4 | thioesterase superfamily member 4 | 1.52 |
|  | ZBTB1 | zinc finger and BTB domain containing 1 | 1.73 |
|  | PRR36 | proline rich 36 | 0.64 |
|  | SNORD108 | small nucleolar RNA, C/D box 108 | 1.65 |
|  | EXOG | endo/exonuclease (5'-3'), endonuclease G-like | 1.28 |
|  | TSPYL2 | TSPY-like 2 | 1.53 |
| **Boys (p-value < 0.0001)** |  |  |  |
|  | ZBTB45 | zinc finger and BTB domain containing 45 | 0.80 |
|  | SBDS | Shwachman-Bodian-Diamond syndrome | 1.70 |
|  | C22orf29 | chromosome 22 open reading frame 29 | 0.76 |
|  | HSP90AA2P | heat shock protein 90kDa alpha (cytosolic), class A member 2, pseudogene | 1.51 |
|  | RBM20 | RNA binding motif protein 20 | 0.46 |
|  | HSP90AB1 | heat shock protein 90kDa alpha (cytosolic), class B member 1 | 1.36 |
|  | KLHL34 | kelch-like family member 34 | 0.61 |
|  | ST13 | suppression of tumorigenicity 13 (colon carcinoma) (Hsp70 interacting protein) | 1.44 |
|  | SNORA5B | small nucleolar RNA, H/ACA box 5B | 0.53 |
|  | ROBO2 | roundabout guidance receptor 2 | 0.50 |

FC: fold change calculated for an increase in PM_2.5_ of 5 µg/m^3^.

# Table S4. Significant differentially expressed genes by short-term PM_2.5_ exposure in cord blood of girls and boys.

| **Gene symbol** | **Gene name** |
| --- | --- |
| **Up-regulated genes for both sexes** | |
| ANKRD44 | ankyrin repeat domain 44 |
| ARMC8 | armadillo repeat containing 8 |
| ATF4 | activating transcription factor 4 |
| ATP6AP2 | ATPase, H+ transporting, lysosomal accessory protein 2 |
| C1GALT1 | core 1 synthase, glycoprotein-N-acetylgalactosamine 3-beta-galactosyltransferase 1 |
| CHD9 | chromodomain helicase DNA binding protein 9 |
| E4F1 | E4F transcription factor 1 |
| EDRF1 | erythroid differentiation regulatory factor 1 |
| EIF1 | eukaryotic translation initiation factor 1 |
| IL6ST | interleukin 6 signal transducer |
| LARP1 | La ribonucleoprotein domain family, member 1 |
| MBD1 | methyl-CpG binding domain protein 1 |
| NBPF9 | neuroblastoma breakpoint family, member 9 |
| PBRM1 | polybromo 1 |
| RPS25 | ribosomal protein S25 |
| SRSF1 | serine/arginine-rich splicing factor 1 |
| SRSF11 | serine/arginine-rich splicing factor 11 |
| UBE2K | ubiquitin-conjugating enzyme E2K |
| UTRN | Utrophin |
| VPS13C | vacuolar protein sorting 13 homolog C (S. cerevisiae) |
| **Down-regulated genes for both sexes** | |
| APBB2 | amyloid beta (A4) precursor protein-binding, family B, member 2 |
| CACTIN | cactin, spliceosome C complex subunit |
| CNTNAP1 | contactin associated protein 1 |
| CYP4F62P | cytochrome P450, family 4, subfamily F, polypeptide 62, pseudogene |
| DOCK3 | dedicator of cytokinesis 3 |
| DRAP1 | DR1-associated protein 1 (negative cofactor 2 alpha) |
| GUF1 | GUF1 homolog, GTPase |
| HAMP | hepcidin antimicrobial peptide |
| HIC1 | hypermethylated in cancer 1 |
| HMG20B | high mobility group 20B |
| IL2RG | interleukin 2 receptor, gamma |
| KCTD19 | potassium channel tetramerization domain containing 19 |
| KRT7 | keratin 7, type II |
| KRTAP1-3 | keratin associated protein 1-3 |
| KRTAP2-4 | keratin associated protein 2-4 |
| LINC00320 | long intergenic non-protein coding RNA 320 |
| MAP1S | microtubule-associated protein 1S |
| MTRNR2L10 | MT-RNR2-like 10 |
| NACAP1 | nascent-polypeptide-associated complex alpha polypeptide pseudogene 1 |
| NUTM2D | NUT family member 2D |
| PCSK1N | proprotein convertase subtilisin/kexin type 1 inhibitor |
| PFKL | phosphofructokinase, liver |
| PIK3CD-AS1 | PIK3CD antisense RNA 1 |
| PRR25 | proline rich 25 |
| PRR36 | proline rich 36 |
| SFTPA2 | surfactant protein A2 |
| TGFA | transforming growth factor alpha |
| TMEM151B | transmembrane protein 151B |
| TOMM20L | translocase of outer mitochondrial membrane 20 homolog (yeast)-like |
| TSHB | thyroid stimulating hormone, beta |
| TSPAN11 | tetraspanin 11 |
| ZNF205 | zinc finger protein 205 |
| ZNF771 | zinc finger protein 771 |
| **Down-regulated genes for boys, up-regulated for girls** | |
| CECR5-AS1 | CECR5 antisense RNA 1 |
| KDM5D | lysine (K)-specific demethylase 5D |

Table S5. Top ten significant genes in cord blood of newborn boys and girls associated with short-term PM_2.5_ exposure.

| **Sex** | **Gene symbol** | **Gene name** | **FC** |
| --- | --- | --- | --- |
| **Girls (p-value < 0.0026)** |  |  |  |
|  | ASTN2 | astrotactin 2 | 0.84 |
|  | THOP1 | thimet oligopeptidase 1 | 1.11 |
|  | CDC16 | cell division cycle 16 | 1.22 |
|  | MRPS2 | mitochondrial ribosomal protein S2 | 1.12 |
|  | ZNF404 | zinc finger protein 404 | 1.30 |
|  | TRIM61 | tripartite motif containing 61 | 1.21 |
|  | PRP36 | proline Rich Protein 36 | 0.75 |
|  | MRPS25 | mitochondrial ribosomal protein S25 | 1.15 |
|  | NACAP1 | nascent-polypeptide-associated complex alpha polypeptide pseudogene 1 | 0.72 |
|  | SRSF6 | serine/arginine-rich splicing factor 6 | 1.19 |
| **Boys (p-value < 0.0014)** | | | |
|  | MTRNR2L3 | MT-RNR2-like 3 | 0.75 |
|  | AVL9 | AVL9 homolog (S. cerevisiase) | 1.30 |
|  | TNP2 | transition protein 2 (during histone to protamine replacement) | 0.82 |
|  | MTRNR2L7 | MT-RNR2-like 7 | 0.78 |
|  | ADAM11 | ADAM metallopeptidase domain 11 | 0.73 |
|  | POTEF | POTE ankyrin domain family, member F | 1.17 |
|  | ITGB1 | integrin, beta 1 (fibronectin receptor, beta polypeptide, antigen CD29 includes MDF2, MSK12) | 1.14 |
|  | NRG4 | neuregulin 4 | 0.69 |
|  | MTRNR2L10 | MT-RNR2-like 10 | 0.76 |
|  | HYALP1 | hyaluronoglucosaminidase pseudogene 1 | 0.63 |

FC: fold change calculated for an increase in PM_2.5_ of 10 µg/m^3^.

# Figures

#
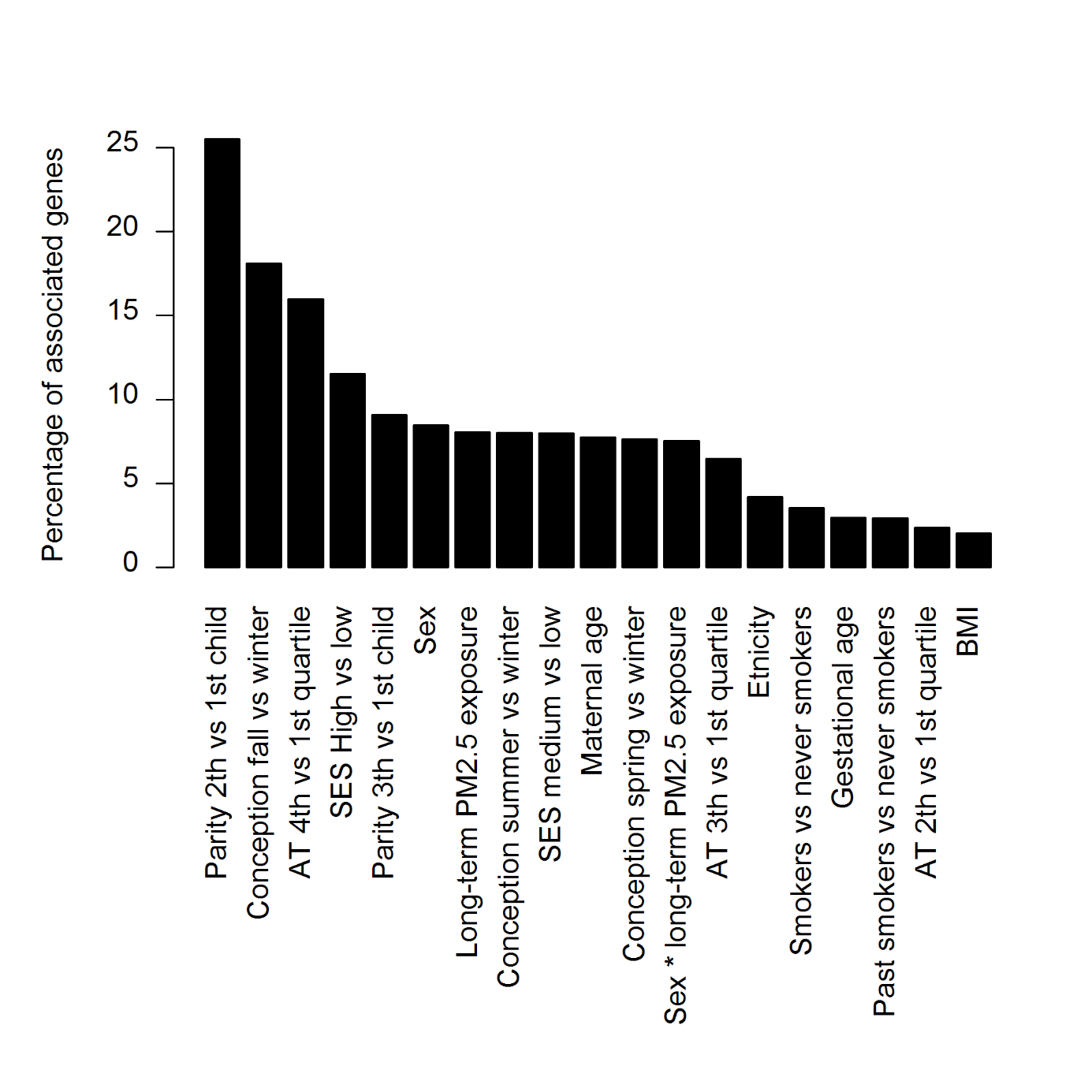
Figure S1. Histogram representing the percentage of genes with p-value <0.05 for each variable included in the model.

#
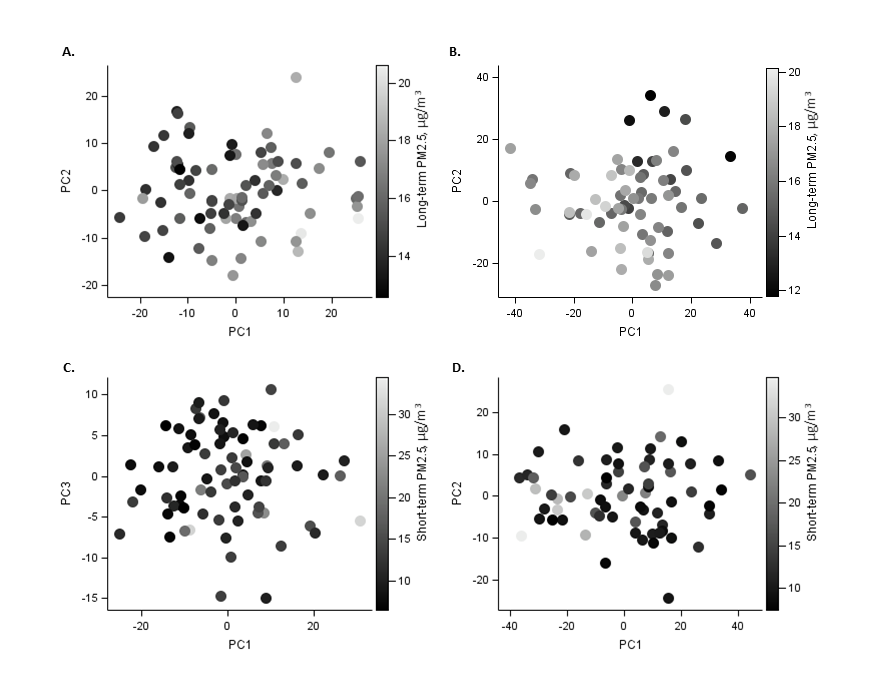
Figure S2. Principal component analysis plot showing the transcriptomic response to long- and short-term PM_2.5_ exposure in *(A, C)* girls and *(B, D)* boys.

The plot is based on the PM_2.5_ modulated genes (p-value<0.05). A color gradient (dark-light) represent the level of PM_2.5_ exposure (low-high). PC: principal component.


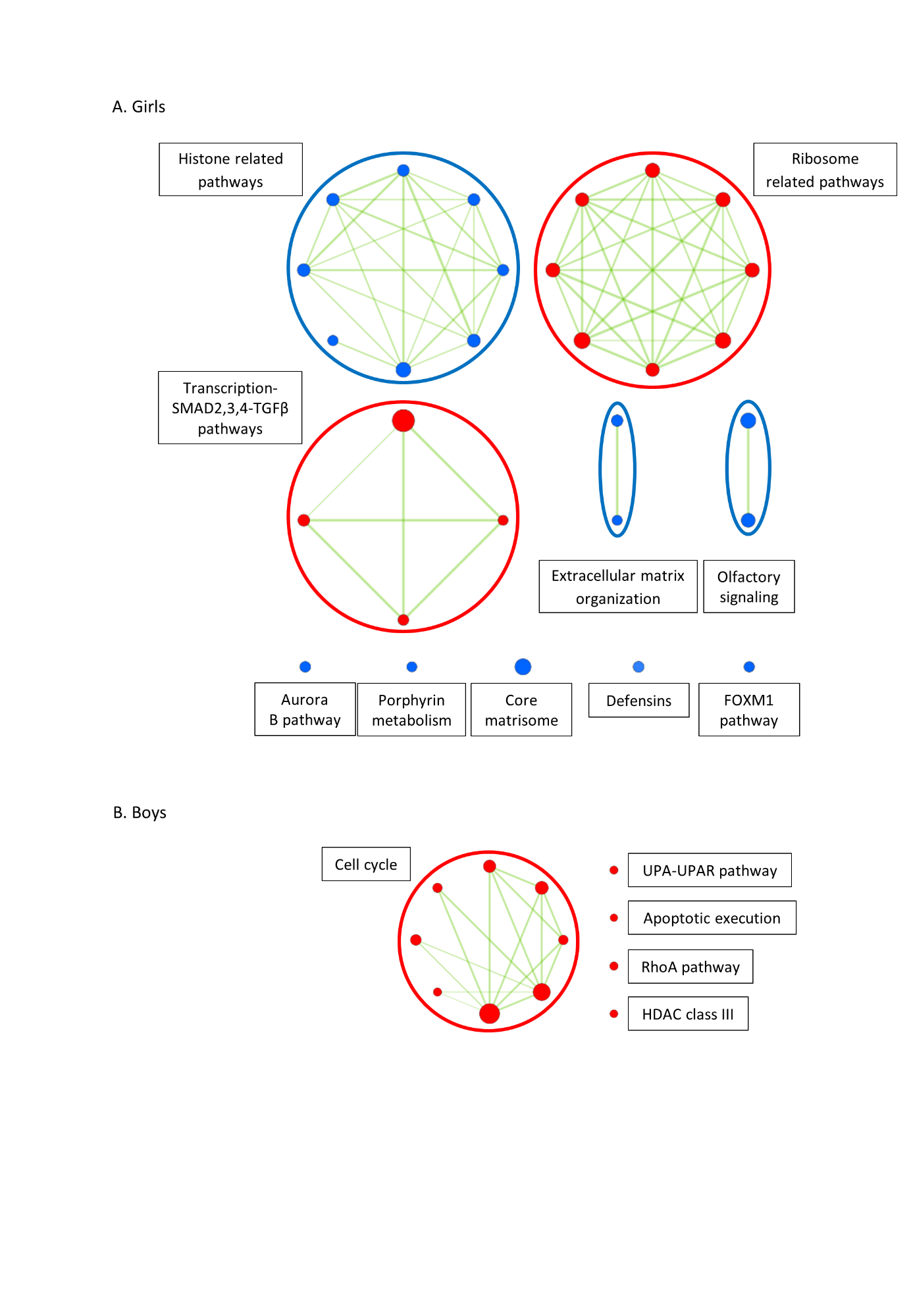
Figure S3. Pathways modulated by long-term PM_2.5_ exposure for girls (*A*) and boys (*B*) resulting from GSEA.

The size of the nods represents the size of the pathway. Related pathways are encircled, assigned a label, and connected by green lines, representing common genes between pathways. Up-regulated and down-regulated pathways are given in red and blue, respectively. uPAR: Urokinase-type plasminogen activator (uPA) receptor, HDAC: histone deacetylase, FOXM1: forkhead box M1, RhoA: Ras homolog gene family member A.


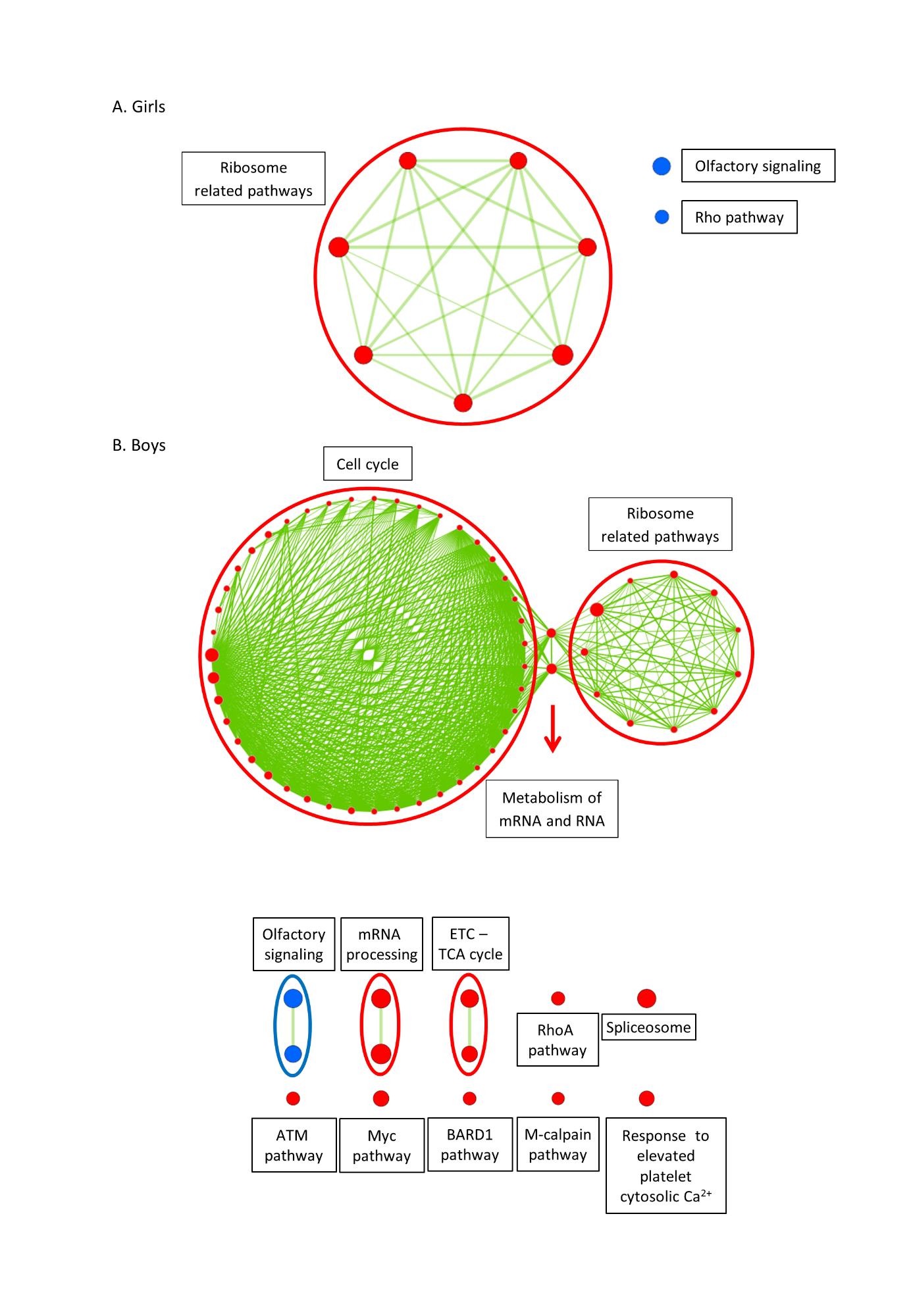
Figure S4. Pathways modulated by short-term PM_2.5_ exposure for girls (*A*) and boys (*B*) resulting from GSEA.

The size of the nods represents the size of the pathway. Related pathways are encircled, assigned a label, and connected by green lines, representing common genes between pathways. Up-regulated and down-regulated pathways are given in red and blue, respectively. Rho: Ras Homolog gene family, TCA: tricarboxylic acid, ETC: electron transport chain, ATM: Ataxia Telangiectasia Mutated, BARD1: BRCA1 associated RING domain 1. Myc: v-myc avian myelocytomatosis viral oncogene homolog.

**References**

Cox B, Martens E, Nemery B, Vangronsveld J, Nawrot TS. 2013. Impact of a stepwise introduction of smoke-free legislation on the rate of preterm births: Analysis of routinely collected birth data. Bmj 346:f441.
